# Male rape: survivors, support and the law in late twentieth-century England and Wales

**DOI:** 10.1093/hwj/dbae002

**Published:** 2024-04-11

**Authors:** George J Severs

**Affiliations:** Geneva Graduate Institute, Switzerland

**Keywords:** sexual assault, psychiatrists, legislation, reform, support, activism

## Abstract

Until 1994, men were not recognized legally as victims of rape in England and Wales. This article explores the history of male survivors of rape there, establishing the uneven patchwork of support services available to them prior to 1994. It argues that a growing psychiatric literature which studied male survivors of sexual violence was a major factor in convincing lawmakers to include men as potential victims of rape in updated sexual offence legislation. Other medical professionals played key roles in bringing male survivors to police attention, but psychiatric research was most influential in changing the policy agenda in this arena.

**Figure 1:**
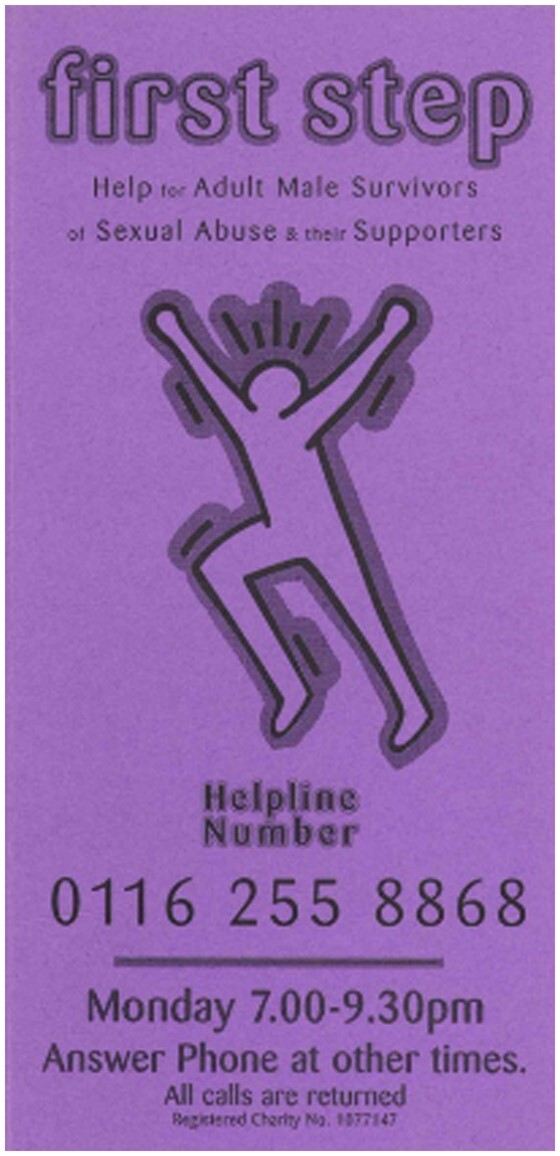
First Step leaflet, 2001. Reproduced with permission of First Step.

In June 1995, Andrew Richards was sentenced to life for attempted rape. His ‘long history of sex crimes’ influenced the particularly long sentence, with the judge imposing a minimum ten-year custodial term. It was not just his long sentence or sexually violent history which drew journalists to Richards. He had become the first man convicted of (attempted) rape of another man. The newly enacted Criminal Justice and Public Order Act 1994 had expanded the definition of rape to include both male and female victims. Ernest Woollett, acting as a spokesperson for Survivors, a London-based support group for male victims of rape, told the BBC that ‘the signal it sends out to men is that this is now being taken seriously and that the powers-that-be are prepared to take it seriously’.^1^

Woollett’s remark points to a history of male survivors who had experienced disbelief, silence and isolation following their rapes. It also suggests that a change in rape law had long been on the agenda of activists and professionals working with male survivors of sexual violence. This article explains why male rape victims began to be ‘taken seriously’ by the criminal justice system in England and Wales in the mid-1990s. New understandings of male trauma, the impact of psychiatric studies of male rape survivors, and a growing landscape of support organizations all speak to areas of significant change in the ways male rape was understood in late twentieth-century Britain. The change in rape law in England and Wales in 1994 was just one part of this shifting landscape, but attending to that legal shift makes visible these less tangible cultural shifts, which, in turn, facilitated such legislative reform.

In the years preceding the enactment of male rape legislation, the British state was not uninterested in regulating male sexual conduct, especially where that conduct related to sex with other men. Sodomy was a capital offence from 1533 until 1861, but as with the sex (or species) of those involved, consent was not taken into account.^2^ Indeed, such legislation was more invested in ‘safeguarding civility’ than guarding against unwanted sex acts.^3^ Nineteenth-century jurists utilized legislation against sodomy and buggery for the purpose of ‘protecting public morality’; it was the fact that two men were engaged in a sex act (very few cases were brought concerning the buggery of a woman or an animal) which concerned them, rather than a potential lack of consent.^4^ Whilst this forms part of a longer history of regulating sex between men, this article focuses on the emergence of legislation which specifically prohibited non-consensual sex in which a male person figured as a victim. The terminology used to discuss those subjected to non-consensual sex (both men and women) can be contentious. I deploy the term ‘survivor’ as well as ‘victim’ when discussing men who have been raped by other men. Whilst I am sympathetic to the historian Joanna Bourke’s ambivalence about the politics of the term ‘survivor’, I have opted to use it throughout because it reflects the language used and identities articulated by those in my sources (such as Woollett’s group Survivors). As many of those in this article were fighting to be seen in law as *victims* of a specific crime, I also use the term victim here.^5^

Individual cases abound in discussions of male rape, notably the recent and high profile example of Reynhard Sinaga. Sinaga remains the most prolific convicted rapist in British legal history, convicted in 2020 of raping or sexually assaulting forty-eight men but believed by Greater Manchester Police to have committed sexual offences against over 200.^6^ These highly publicized cases can suggest that sexual assaults committed against men are unusual. In fact, a report published in 2022 suggested that half of all men have experienced ‘unwanted sexual experiences’, forty-two per cent ‘have experienced at least one sexual crime, as legally defined’, and one in ten men have been subjected to rape ‘or non-consensual penetration’ in England and Wales.^7^ Far from being abnormal or an aberration, male rape is widespread, and survivors are a social and cultural reality whose experiences require historicizing.

This article explores the changes to male rape law in the late twentieth century, arguing that psychiatric understandings of male survivors of rape, which developed throughout the 1980s and 1990s, were a crucial catalyst for this change. To account for the changing levels of socio-legal visibility male survivors of rape experienced in the late twentieth century, this article begins by examining the threadbare patchwork of support available to them before legal recognition. It looks at Rape Crisis Centres in the 1980s, identifying increasing but small scale and uneven levels of support available to male rape victims during the 1980s and into the 1990s. Queer voluntary organizations were more likely to offer guidance, referrals and support to men who had been raped, but heterosexual survivors were often reluctant to access services which were coded as ‘gay’.

Having established that support for male survivors was limited in the years before 1994, this article argues that psychiatric studies were catalysts for the growing visibility of male survivors, especially amongst legislators and policymakers. As I show, medical professionals were often key to referring men who had been raped to the police, thus making them visible to the criminal justice system, while a growing psychiatric literature rendered male survivors legible as both larger in number and more psychologically affected by sexual violence than many legislators had thought previously. Drawing on some of the key contributions to psychiatric research on male rape in the late 1980s, I use Home Office documents and parliamentary debates to demonstrate their impact on changing legal definitions of rape in the mid-1990s.

Examining the history of male rape in late twentieth-century Britain not only begins to account for a significant legal change and the resultant provision of services for male victims of sexual violence; it also reveals the ways in which masculinity was being reinterpreted during this period through developing understandings of trauma. As I will show, the literature on trauma, particularly post-traumatic stress disorder (PTSD), disrupted conventional models of masculinity by rendering visible the interior psychic injuries inflicted upon raped men. Through an investigation of new understandings of, and responses to, male rape victims, this article offers fresh perspectives on late twentieth-century masculinities.

The history of rape and sexual violence has been well researched, and male victims have not been absent from these studies. The most famous line of Susan Brownmiller’s *Against Our Will* (1975), in which she defines rape as ‘a conscious process of intimidation by which all men keep all women in a state of fear’ often overshadows the presence of male victims of rape (especially gay men and prisoners) in her analysis.^8^ Joanna Bourke’s numerous historical studies of rape and sexual violence have also attended to male victims.^9^ Most recently, Bourke has surveyed the multiple cultural, legal and linguistic barriers to male survivors disclosing their abuse across several global case studies.^10^

Despite these contributions, as Bourke notes, raped men have been ‘relegate[d] … to a dismissive footnote’ within the wider historiography.^11^ Looking at the history of male victims of sexual abuse in colonial India, Deana Heath suggests three reasons that such experiences have been historically neglected or ‘hidden’: a lack of legal acknowledgment, accompanied by silence from male victims (enforced by legal and gendered structures), which coalesce to normalize models of sexual violence in which men are ‘framed primarily as perpetrators and women as victims’.^12^ A similar lack of legal acknowledgment in England and Wales up to 1994 produced a historical framework which excluded male victims from histories of rape and sexual offences.^13^ Addressing this historiographical gap, this article examines the ways in which legal recognition of male rape in England and Wales was prompted by activists and emerging psychiatric understandings of the traumatic impact of such experiences. It argues that shifting psychiatric and cultural ideas about masculinity in the late twentieth century made male rape newly legible.

I follow Heath’s argument that, far from being ‘exceptional or aberrant, sexual violence against men is … part of an array of institutionalized and socially sanctioned violence that serves to empower certain groups of men as heterosexual, masculine, and dominant’.^14^ In other words, this is a feminist project. Patriarchy and rigidly gendered notions of masculinity have prevented male victims of rape from speaking about their experiences for decades. This history of male survivors does not seek to displace female survivors, or to establish a ‘hierarchy of suffering’ (which Bourke rightly warns will ‘endorse … some kinds of abuse’).^15^ Rather, it seeks to understand the ways in which changing understandings of masculinity made male survivors more visible, and points to a feminist, trauma-informed gender politics to aid this process.

This article examines several English case studies and the law in England and Wales, but male rape was not an issue affecting only these parts of Britain. Historians of the British imperial project have been particularly attentive to the introduction of male rape legislation, and the utilization of rape against men as a systematic tool of torture, making it all the more curious that historians of the metropole’s sexually violent histories have been so reticent to deal with male rape.^16^ Male rape was being reconsidered across the four nations of the United Kingdom in the late twentieth century. In November 1991, the Lesbian, Gay and Bisexual Network and the Scottish Homosexual Rights Group hosted a conference at which ‘male rape’ was listed as one of the ‘topics for discussion’ by the assembled activists.^17^ In Belfast, there is evidence that the Rape Crisis Centre was engaged in HIV/AIDS related training for their volunteers which may have involved thinking about male as well as female rape survivors.^18^ Outside the UK, in the Republic of Ireland, complaints made to An Garda Síochána by male sexual assault victims more than doubled between 1994 and 1995.^19^ England and Wales, however, was the first UK jurisdiction to include men in its definition of rape. As such, it offers a unique vantage point through which to examine the emerging legal and cultural image of the male rape survivor.

In what follows I focus on male rapes committed by other men. Not all male survivors are raped by men, but most are.^20^ This is not to minimize the experiences of men and women who have been raped by women. I examine ‘male rape’ through the definitional boundary of men raped by other men not only because these were the overwhelming majority of cases reported in the press and to various state and voluntary agencies. I do so also because the law, both before and after it was updated to include male victims in 1994, defined the rapist as male. Indeed, it remains the case in England and Wales that only men are *legally* capable of committing rape.^21^

Finally, I focus my attention on adult male survivors of rape as distinct from male children subjected to sexual abuse. Victims of child sexual abuse became increasingly visible across the period discussed in this article. Huge public concern following an unprecedented rise in the diagnosis of child sexual abuse in the Cleveland area of North Yorkshire in 1987 led to a high-profile inquiry, whilst the National Commission of Inquiry into the Prevention of Child Abuse made visible ‘hundreds of adult survivors of child sexual abuse’ during the mid-1990s.^22^ An unequal age of consent for gay men resulted in an unequal risk of committing a sexual offence against a child in law (discussed below) which complicated this historical picture. However, men raped as adults and child victims of sexual abuse suffered particular experiences which were legislated against distinctly, hence their separation in my analysis.

## MALE SURVIVORS AND RAPE CRISIS CENTRES BEFORE LEGAL RECOGNITION

By the early 1980s, rape crisis centres were well established across Britain, having been founded by women’s liberation activists throughout the 1970s.^23^ These had been set up as ‘by women, for women’ centres, but some did engage with men. In March 1983, for example, volunteers from Birmingham Rape Crisis and Research Centre (BRCRC) spoke at a meeting of the Gay Youth Group in Handsworth.^24^ The content of the meeting is not recorded, but by engaging with young queer people about sexual violence, BRCRC were beginning to see gay men (as well as lesbian women) as real and potential victims of rape by men in the early 1980s.^25^ The group appeared at events with the local Men Against Sexism group, part of a late twentieth-century anti-sexist men’s movement for whom ‘rape became a haunting presence’.^26^ As Lucy Delap has argued, feminist thinking about rape ‘forced some men into useful re-evaluations of their “sex lives”’, and this event appears to show this re-evaluation in action.^27^ BRCRC remained a centre which catered for female rape survivors during this period, but throughout the 1980s they – and other rape crisis centres – increasingly encountered male survivors.

Some rape crisis centres’ helplines took calls from men and offered some services for male survivors, but the need to safeguard the partners of abusive men, and dealing with endemic levels of physical and sexual violence against women, meant it was often practically and politically difficult for rape crisis centres to accommodate men.^28^ One clinical psychologist who was involved in the London Rape Crisis Collective (LRCC) during the late 1970s and 1980s, for example, recalled that the group ‘didn't counsel men who had been raped’, a position which was ‘very powerful, I think’ because ‘it was a service by women for women’.^29^ For this psychologist and many feminist campaigners, running services on these lines was empowering and necessary. The men who had called them, many of whom will have known this was a women’s service, were left with few options of help. Two research psychiatrists working on male rape in the late 1980s suggested that ‘the politicization of rape as a feminist issue may contribute to the isolation and suffering experienced by the male victim’.^30^ That politicization was a perfectly rational one based on the scale of the problem of male physical and sexual violence committed against women and rape crisis centres’ lack of resources. Such dire straits constricted the number of people able to access their help, leaving women’s rape crisis centres with little choice but to refuse to provide services to men.

This sense of isolation was difficult for many men who wanted to be recognized as survivors of rape. Of course, not all male survivors did understand themselves in such terms. Psychiatric studies made clear the high levels of personal confusion and identity crises which many survivors – men as well as women – suffered from.^31^ The forces of shame and stigma, combined with the limited availability of specialist services for male survivors, isolated many raped men and kept them from seeking help.^32^ The few services which were available to male survivors of sexual violence were often explicitly coded, or understood by male rape survivors, as being for gay men. LRCC would often refer their male callers to London Friend, which had operated as the ‘befriending and counselling arm’ of the Campaign for Homosexual Equality since 1971.^33^ Volunteers at LRCC understood London Friend to be a ‘gay male counselling service’.^34^ Some men may have found this a useful referral but, as the clinical psychologist who worked with LRCC remembered, ‘a lot of the men said, you know, but I’m not gay and so I don’t want to ring a gay male counselling service’.^35^ Male rape survivors who did not identify as gay were often left without support services by both the state and the wider rape crisis movement throughout the late twentieth century. Given the tendency for such survivors to suffer sexual identity crises, this level of isolation was not only personally unsatisfactory but also psychologically detrimental.

## QUEER SUPPORT

Many queer activists avoided the subject of male rape during the 1970s and were reluctant to discuss the emerging reality that sexual violence existed among gay men. Such avoidance is attributable to what Bourke describes as ‘justifiable fears that evidence of sexual violence within their community would be annexed to homophobic agendas’.^36^ Referrals to London Friend, however, reveal the existence of a network of queer support services which were responding to male rape survivors from the late 1970s onwards.

Gay Switchboard (Lesbian and Gay Switchboard from 1986) was one such organization.^37^ Founded in London in 1974, Switchboard offered a helpline for lesbians, gay men and their kin. Many would call with legal concerns or questions which were subsequently recorded in Switchboard’s ‘legal logs’. Calls about ‘rape/incest’ were rare, but several such records do exist. On 21 March 1992, for example, a man called from a ‘police rape suite’ having been ‘raped by [his] ex-lover’.^38^ Other calls recorded by Switchboard suggest an alternative way in which ideas about male rape figured in the minds of gay male callers. The age of consent for male homosexuals was set at twenty-one until 1994 when it was reduced to eighteen, both higher than the heterosexual standard of sixteen. In other words, sex with young men aged between sixteen and twenty-one (latterly changing to eighteen) was a sexual offence until January 2001, when the age of consent was equalized. There was thus a legal threat of rape for older men who were involved in sexual relationships with those still considered ‘minors’. In one complicated case from 1992 (when the age of consent for gay men was twenty-one), a man called Switchboard after his eighteen-year-old lover had stabbed him. The caller was due to appear ‘as a police witness’ in the case but was ‘worried he will be charged as a result for having sex with a minor’.^39^ Similar anxieties motivated several older men who had ‘relationships’ with men under the legal age of consent, forcing them to explain to Switchboard volunteers that ‘the sex was very definitely consenting.’^40^ A complex and counter-intuitive legal landscape therefore made it difficult for men to call Switchboard (and medical or legal authorities) about both rape and sexual matters: where forced sex between men had occurred, the law did not recognize this as rape; where sex was not forced, but took place between men either side of the unequal age of consent, the state could see this as a sexual offence. The brevity of the logs’ notes makes it difficult to draw out conclusions about prevalence of male rape or whether ‘male rape’ was a broadly used (or usable) term amongst Switchboard volunteers. What the legal logs do reveal, however, is a limited but extant patchwork of organizations to which male survivors could be referred.

The main organizations to which callers concerned about sexual violence were referred were Gay Legal Advice (GLAD) and the Gay London Police Monitoring Group (GALOP).^41^ Both were small organizations with broad remits to offer advice and support to queer people navigating aspects of the criminal justice system. Small budgets and increasing demand meant that they were unable to support all the male survivors who contacted them. One man who had been raped in London 1992 called Switchboard after refusing medical attention. He was referred to ‘GALOP in lieu of GLAD’; it seems that Switchboard could not get through to anyone at GLAD that evening, a neat but frustrating example of the precarious and limited options of support open to male survivors of rape.^42^

The Switchboard legal logs do not provide much detail of the incidents callers presented to volunteers, but they do begin to reveal the fact that charities and support groups were willing to deal with calls concerning male rape and sexual assault. These were far from ideal avenues. As we have seen, some survivors were unable to access these helplines. Whilst GLAD and GALOP were able to take Switchboard referrals from male survivors of rape and sexual assault, as well as accused perpetrators, neither offered dedicated advice solely on this issue.

## MALE SURVIVOR GROUPS

There was, however, a small number of support groups for male survivors of rape and sexual violence. These had existed since the mid-1980s, and by the time the calls discussed above were coming into the Switchboard helpline the number of male survivor groups was growing across England.

Survivors, a group established in London by Martin Dockrell and Richie McMullen in 1986, was perhaps the most well-known support group for raped men. This was due in part to the publication in 1990 of McMullen’s book *Male Rape: Breaking the Silence on the Last Taboo*.^43^ Published by Gay Men’s Press, the book was read widely by the nascent male survivors movement and was available in radical and queer bookshops across the country.^44^ As one of the few charities offering support and advice for male victims of rape, the man who had called Switchboard from a police rape suite in 1992 was given Survivors’ number.^45^ Other male rape victims might have encountered Survivors if they were taken to St Mary’s Hospital in Paddington, where they operated a male rape counselling service.^46^ Survivors also ran ‘support groups at Colindale Hospital’ in the early 1990s.^47^ The group took inspiration from Survivors Sheffield, which had been founded in 1984, two years before the London group. Survivors Sheffield defined their organization as ‘a voluntary group who have counselled male survivors of sexual abuse and rape, and male partners of survivors’, as well as operating a telephone helpline and facilitating educational workshops.^48^

McMullen’s Survivors group in London also ran a telephone helpline, and data on the number of calls received across its first year (1988) begin to reveal the demand for tailored support for male rape survivors. Calls increased rapidly throughout 1988, and by December the helpline was receiving five times the number of calls it had taken in January.^49^ This number appears to have grown dramatically year on year as the organization became more prominent and, as we shall see, understandings of the impact of sexual violence on men became more nuanced and widespread. In 1992 Survivors was receiving around 800 calls a year, and by 2002 this had risen to 5,138 calls.^50^

Before long, Survivors had a growing presence across the UK. Anti-sexist men’s magazines advertised groups like Survivors Sheffield and publicized calls to form new local groups.^51^ In 1991 John Hogget, an anti-sexist and male rape activist based in Reading, wrote to the anti-sexist men’s magazine *Men for Change* to share his conviction that ‘there should be a crisis telephone line in Reading catering for men and boys who are being, or have been, sexually abused or raped’.^52^ Hogget subsequently founded a self-help group for male survivors of sexual assault in Reading and by 1992 was addressing major male survivors’ conferences.^53^ As this suggests, activist and support groups for male survivors in Britain were unevenly but increasingly visible throughout the course of the 1990s.

## HIV/AIDS, MEDICS AND THE POLICE

Despite this growth, male survivor groups remained rare in Britain. Male survivors were more likely to turn to medical professionals for support in the first instance, as opposed to the police or voluntary organizations. In particular, genitourinary medics were key channels between male survivors of sexual assault and the police. Homophobia, both perceived institutional police homophobia and the internalized homophobia of some survivors, was a major force preventing survivors from reporting their experiences to the police.^54^ There were also more practical obstacles preventing police forces from dealing with male survivors. Until November 1990, men were barred from being examined in the Metropolitan Police Service’s victim examination suites.^55^ Police surgeons and forensic medics were thus unable to medically examine men reporting rape and sexual assault and were prevented from completing rape kits in these cases.

Even after this policy changed, some male survivors were not seen by police surgeons. One man who was raped in 1992 by a taxi driver in central London was taken by police officers (almost certainly Metropolitan Police Officers) ‘to St Mary’s casualty’ instead of to a victim examination suite. In this case, the officer’s decision meant that no evidence was collected. Feeling ‘too embarrassed to hang around’ the survivor left ‘casualty’ and travelled home ‘in a cab’ before calling Switchboard for advice.^56^ This case is an important reminder that male survivors continued to be overlooked by legal and medical authorities even after official policy changes designed to include them were enacted.

The ongoing HIV/AIDS epidemic not only had an impact on the concerns of male survivors during the 1980s and 1990s, but also influenced their willingness to seek medical attention. Following an instance of sexual violence, male victims frequently worried about possible exposure to HIV. Unlike many people in Britain, whose worries about the virus were caused by media and politically induced hysteria, male survivors’ concerns were often well placed.^57^ One study of male rape survivors across the UK published in the early 1990s found that fifty-seven per cent of assailants had used the threat of HIV transmission during the assault. Follow-up tests concluded that eighteen per cent of cases had resulted in the transfer of a sexually transmitted disease.^58^

Survivors’ fear of HIV was often what brought them to the attention of medical professionals in the first instance. In the early 1990s it was often medics who were the first to have instances of sexual assault ‘reported’ to them, rather than police referring men to sexual assault referral centres (SARCs), as is more common today. Two consultants working in sexually transmitted disease clinics in London reported concern ‘that they were seeing an increasing number of men asking for AIDS testing at the clinic who claimed that they had been assaulted but who refused or did not want to inform police of their attack’.^59^ Elsewhere, an article by genitourinary medics working at St. Mary’s Hospital in London and the Edinburgh Royal Infirmary reported (amongst others) the case of a sixteen-year-old gay man who ‘initially presented with a request for HIV antibody testing’ in 1986. It was only after asking this young man more questions (‘taking a full history’) that they discovered ‘his only sexual encounter was two years previously’ when he was violently raped by a man living with AIDS.^60^

As I argue below, developing ideas of male trauma made male survivors of sexual violence more visible to, and understood by, elements of British society, especially lawmakers. This was not a teleological development. Instead, it coincided with a significant rise in anxieties about, and stigmatization of, sex between men because of associated fears of HIV/AIDS. Press reports about male rape in the early 1990s were published at the height of the British epidemic: 1995 alone saw 1,500 new HIV diagnoses and more than a thousand AIDS-related deaths.^61^ The following year, the UK AIDS-related death toll exceeded 12,000.^62^ Discussions of male rape, therefore, occurred as part of a wider renegotiation of sexual mores which the epidemic had engendered. As Matt Cook has demonstrated, whilst the HIV/AIDS crisis elicited multifaceted and uneven responses, it certainly hardened anti-gay sentiment. In 1987, the British Social Attitudes Survey reported that 74 per cent of its respondents agreed that ‘homosexual relations are always or mostly wrong’.^63^ Typical of this AIDS-induced homophobia was the belief that HIV-positive people had ‘brought it on themselves’.^64^ As HIV/AIDS was commonly seen as a ‘gay plague’, just as male rape was thought of frequently as a ‘homosexual offence’, the epidemic went some way in creating an environment which was unsympathetic to male survivors of rape.^65^ If AIDS could be viewed as self-inflicted by ‘promiscuous’ or ‘sexually immoral’ queer sex, so too could male rape, especially when the victims were gay men. This attitude was not hegemonic, as we shall see, but it has proved pervasive and enduring. As recently as June 2022, a Conservative MP told a young gay man who had complained to her of being groped by Prime Minister Boris Johnson’s deputy chief whip Chris Pincher that his homosexuality ‘didn’t make it [his complaint] straightforward’, speaking to a common rape myth that gay men are hyper-sexual, unrapable or ‘asking for it’.^66^

Those sympathetic to male rape law reform sometimes conjured the threat of HIV/AIDS as part of their argument for changing the law. In a 1994 article for the *Guardian* newspaper, which was broadly in favour of reforming British rape laws, journalist Clare Dyer reported on a case in which ‘a male victim’ had been ‘infected with the Aids virus’ after being raped. Dyer described his diagnosis as ‘effectively a death sentence’ and compared it to his attacker’s ‘sentence of only six years’, arguing that without the increased sentencing power which a rape (rather than indecent assault) conviction allowed, this sentence was disproportionately low.^67^ This formed part of a wider discourse about criminalizing the transmission of HIV in the UK.^68^ Such calls were not directly successful, but judgments in 2004 and 2005 determined that transmitting HIV intentionally or recklessly could be prosecuted as grievous bodily harm (GBH).^69^ These later judgments also called into question whether failing to disclose one’s HIV status vitiated consent to engage in sexual acts; but in cases such as these, involving HIV transmission, prosecutions have been for GBH offences rather than rape. For much of the 1990s, it was not HIV’s potential to ‘create’ an offence of rape that worried people sympathetic to legal reform. Instead, it was the fact that prosecutions for rape were not possible *despite* the perceived risk of HIV transmission (a perception rooted in the association between HIV/AIDS and sex between men).

Whether it was an exacerbating factor in a ‘rape’ case, or part of a broader anxiety about queer sex, AIDS certainly shaped the discourse about male rape in multifaceted and uncertain ways. Prejudice and homophobia, alongside genuine concerns about contracting the virus, led many to understand sex between men within a framework of fear and shame. HIV/AIDS activists focused mainly on consensual queer sex in their sex-positive campaigns, in which male survivors of rape sat uneasily.^70^ And yet real anxieties about contracting HIV following an act of rape led many survivors to disclose their experience to a medical professional. As the cases mentioned above demonstrate, genitourinary or emergency medics were often the people who first recognized men as having survived acts of rape. Research psychiatry would amplify this recognition on a larger scale.

## PSYCHIATRY

In Britain, two forensic psychiatrists were responsible for breaking new ground in researching male survivors of sexual violence. Gillian Mezey and Michael King met in the early 1980s working at the London-based Maudsley Hospital, whose history of treating ‘nervous disorders’ dated back to the First World War.^71^ By the early 1980s, the Maudsley ‘was *the place* to train’ for budding psychiatrists, and it was there that Mezey and King developed the skills and interests which would lead them to careers in forensic psychiatry.^72^

Late twentieth-century psychiatrists were engaged in a process of reassessing the mental injuries inflicted by trauma. In particular, understandings of post-traumatic stress disorder (PTSD) were changing, with the disorder being diagnosed more frequently in domestic settings, rather than solely armed conflict situations. Pioneered by American psychiatrists, domestic understandings of trauma had been developing throughout the 1970s.^73^ King, Mezey and their contemporaries arrived at the Maudsley in 1981, a year after the model for diagnosing PTSD was published.^74^ King and Mezey developed an interest in the traumas resulting from sexual violence, and early British studies focused on female survivors of rape.^75^ As the authors of several of these studies, and as individuals ‘both quite interested in gender politics’, Mezey and King were aware of this focus. ‘We were talking one day about how all of the research on sexual violence was all about women’, Mezey recalled, leading them to ask: ‘what about men?’^76^

A major turning point in the psychiatric literature occurred in 1989 when Mezey and King published a groundbreaking article in *Psychological Medicine*. In a bid to answer the question they had set themselves, ‘what about men?’, Mezey and King had been interviewing male survivors of rape having ‘advertised for men who had experienced sexual violence’.^77^ This was not the first medical or psychiatric contribution to the British literature on male rape. Mezey and King had published an article on ‘male victims of sexual assault’ two years earlier, which had argued that men experienced similar psychological ‘reactions’ to female victims, but greater levels of stigma. Their 1987 article was published in a medico-legal journal and made an explicit argument for legal change. ‘Increased recognition of these assaults is called for’, Mezey and King argued in the article’s abstract, ‘both in law and by victim support agencies, in order to reduce the stigma, encourage reporting and facilitate the enforcement of criminal justice’.^78^ This article broke new ground, but proved less influential than the pair’s later contribution to *Psychological Medicine*. As I show below, the latter found currency with British policymakers, and to date it has been cited over four times as much as their initial 1987 publication.^79^

Due to the impact of their studies and their personal investment in such research, psychiatrists like Mezey and King were seen as allies by queer activists campaigning for legal reform. King, for example, was invited to give one of the keynote addresses to the First Standing Conference on Sexual Abuse of Men in 1992 which was organized by male rape groups and charities, including Survivors co-founder and queer activist Martin Dockrell.^80^ Twenty years earlier, Gay Liberation Front activists had identified psychiatry as one of the major forces of anti-gay oppression. To Gay Liberationists, psychiatrists ‘rarely consult gay people’ and consequently pathologized them as ‘sick’.^81^ Whilst psychiatrists remained ‘almost exclusively heterosexual’ practitioners who had received ‘homophobic’ training (according to King and his colleague Annie Bartlett in 1999), Mezey and King’s work on male rape survivors represented a turning point in the way some queer activists engaged with psychiatry.^82^

Mezey and King’s work was significant in shifting thinking about male rape from ‘an aberration of institutional life’ to ‘a problem in the wider community’.^83^ In other words, male survivors of rape were not only to be found in prisons, boarding schools or the military, but rather across society in general. This finding challenged centuries-old rape myths about the threat of male rape being confined to institutional settings.^84^ The authors used the language of ‘sexual assault’ to reflect the legal reality that rape legislation did not encompass male victims. They also spoke broadly of ‘sexual assault’ to allow for a widely inclusive definition of male survivors of sexual violence. As these were some of the first studies to analyse the psychological impacts of sexual violence on men, the use of an inclusive definition rather than a rigidly legalistic one was important.

Tellingly, the authors confessed that the ‘greatest difficulty of this study was in persuading men who had been sexually assaulted to come forward’.^85^ Perceptions that rape victims were necessarily female, alongside homophobia and rigid notions of masculinity, served to keep men from engaging with researchers. These attitudes also coloured how the men who did engage with Mezey and King thought about their experiences. One heterosexual survivor summed up how he thought about being raped by saying ‘something very dirty has happened to you that nobody believes can happen – if you let it happen you must be queer, if you’re not a queer it can’t have happened’.^86^ Identifying this reticence to engage with researchers, medics and the criminal justice system was important for subsequent scholars and activists, prompting further empirical research and access campaigns.^87^

This heterosexual survivor’s articulation of his rape is an important reminder that victimhood was a complex and ambiguous identity category for male survivors. Heterosexual men often feared that being sexually assaulted negated both their masculinity and their heterosexuality, believing that forced penetration or male same-sex contact pushed them into a queer sexual identity category which was alien to their experiences. Indeed, news reports were often more sympathetic to heterosexual survivors than gay male victims of rape. In part, this was due to an understanding of the impact rape had on masculine identities, and the lack of support for heterosexual survivors, as discussed at the opening of this article. The HIV/AIDS epidemic also had an impact on the ambiguous landscape of survivor identities. Hierarchies of victimhood were commonly understood and articulated, with ‘innocent’ victims such as haemophiliacs and children seen as more worthy of sympathy and support than other victim groups, mostly gay men and intravenous drug users, who were seen as to some degree deserving of their condition.^88^ As I have argued, male survivors of rape experienced their victimhood through a similar cultural hierarchy: gay men were often seen as a hypersexualized group whose ‘lifestyle’ either put them at risk of sexual violence or coded them as predatory would-be attackers capable of sexually assaulting ‘innocent’ heterosexual victims.^89^ Composing a coherent narrative of victimhood in the face of such a rigid cultural hierarchy (and with their own sense of masculinity and sexuality in flux) was a complex psychic task for raped men.

Individual men may have struggled to comprehend their own masculinities post-rape, but Mezey and King’s work was especially significant in constructing a psychiatric picture of men who had experienced sexual violence. Culturally, male survivors were largely invisible, creating a vacuum in which rape myths could abound.^90^ By establishing a psychiatric profile of the ways in which men responded to sexual attacks, these psychiatrists began to fill that vacuum. This was a major break from previous psychological research, which had concluded that evidence of an erection or ejaculation on the part of the victim was proof that rape had not occurred.^91^ Studies from the late 1980s, including those by Mezey and King, demonstrated the seriously harmful effects which rape and sexual violence inflicted on male victims, helping to debunk misunderstandings about male rape and make visible their mental injuries.

Sexual dysfunction was described by Mezey and King as ‘almost a universal theme’ among their participants.^92^ Such ‘dysfunction’ included shutting off sexually for extended periods of time, an inability to perform sexually, and promiscuity. Mezey and King also reported worrying ‘symptoms’ which they described as ‘distressing and disabling’. These included suicidal thoughts and attempts, drug and alcohol abuse and ‘phobic avoidance’.^93^ Mezey and King were not only able to demonstrate that ‘men can be victims of serious sexual assault outside an institutional setting’, which was itself an important step in creating new perceptions of male rape survivors.^94^ More than this, they were able to show the psychiatric impact such attacks were having on men, presenting clinical problems in need of solutions.

These psychiatric contributions were key in shifting government awareness of, and attitudes to, male victims of sexual violence. In the early 1990s, as ministers and civil servants were wrestling over whether including male victims within rape legislation would be too ‘controversial’, interventions from psychiatrists proved particularly resonant. When the House of Commons Library was asked to prepare a reading list on the subject of male rape for the Home Office, nearly half of the list was made up of psychiatric or psychological literature (the other titles were mostly criminological studies of rape in carceral settings).^95^ Moreover, when Dr Zsuzsanna Adler was invited by Mezey and King to contribute to an edited collection on male victims of sexual assault in April 1989, she became a key conduit through which psychological literature on the subject reached the Home Office.^96^ Adler, who was based at the Police Staff College near the Hampshire town of Basingstoke, sought Home Office approval for her contribution, a request which was followed up in detail.^97^ Civil servants in the Home Office took an active interest in the publication, and were even sent a copy of Mezey and King’s 1989 article a year before its publication.^98^ Cutting edge psychiatric research into male victims of rape and sexual assault, then, was informing government thinking on rape legislation from the late 1980s, even if legislative change was to prove slow.

Such scholarship aimed to engender changes in rape legislation. To be sure, this was not the sole aim of research into male survivors of rape, but it is certainly true that some medics became particularly invested in male survivor research through a desire to change what they saw as an unequal and unjust situation. When asked to reflect on the legacy of her work, Mezey replied:

Well we got a change in the law, which is good, but it hasn’t allowed the situation to improve as much as we hoped that we were gunna … start some kind of a … revolution in, you know, how male victims of rape were seen and how they were treated and how the law dealt with perpetrators, but it hasn’t happened …^99^

Much of the pessimism in this response was anchored in what Mezey saw as the failure of the state to invest in male survivor support. As she understood it, the work she and King carried out ‘hasn’t done what I think we hoped it would do which is create … resources to be able to provide men who experience rape with more targeted … treatment, help, support’.^100^ Initially, Mezey had provided training courses at Bramshill police officer training college in Hampshire (presumably through her connection with Zsuzsanna Adler) which, it was hoped, would allow the police to deal with male survivors in a more sensitive and informed manner. Instead, as police numbers and public sector budgets were cut (especially since 2010), Mezey suspected that these training programmes had been sidelined. Indeed, Bramshill ceased operating as the police officer training college in 2015, having been deemed economically unviable by Home Secretary Theresa May.^101^

Whatever the outcome, Mezey and King’s desire to start a ‘revolution’ in the treatment and understanding of male survivors of rape is significant. As Mezey explained, she and King ‘were certainly very aware of the unfairness and inequality and discrimination that was implicit in the law as [it] was phrased at the time’ and that changing this through their research findings was ‘really important’, remaining ‘the most important legacy from the research’.^102^ This attitude not only helps us to understand some of the motivation behind this high-impact psychiatric research, it also points to the current of activism which was threaded throughout work into male survivors of rape.

## LEGAL CHANGE

In 1992, reports that a 25-year-old man had been raped on the London Underground by two men hit the newsstands.^103^ This was a shocking crime for many reasons, including the sex of the victim. The case was discussed in parliament, with Steve Norris (the Minister for Transport in London) describing it as ‘an appalling crime’.^104^ Writing in *Today*, journalist Andrew Penman commented that the assault was ‘particularly shocking because it is a crime few people can have imagined even existed’.^105^ The rape on the London Underground was one of several cases to receive press attention in the early 1990s. Historically, there is little evidence to conclude that there was a meaningful upsurge in male rape at this time, but certainly the early 1990s witnessed a *perceived* increase in such cases, in part due to this increase in media attention.^106^

This assumed rise in cases acted as a catalyst amongst some parliamentarians and policymakers. Harry Cohen, Labour MP for the London constituency of Leyton, took a particular interest in the matter as part of his campaign to reform rape legislation more widely in this period (Cohen ‘took up the issue’ of marital rape in 1990, for example).^107^ Following the tube rape story, Cohen submitted a parliamentary question to the Home Secretary in May 1992, asking if he would ‘lay down guidelines for the counselling and care of male victims of rape and other serious sexual assaults’.^108^ Cohen had also asked the Home Secretary about the number of prosecutions brought for cases in which men had been raped since 1987, with both questions designed to highlight the legislative vacuum in this area and the resultant low rate of prosecution and lack of support for victims.

Cohen’s questions did not convince the Home Office of a need to change the law. Indeed, the Home Office used the small number of convictions for male sexual assault to justify their unwillingness to make changes to rape legislation. A draft letter from Home Secretary Kenneth Clarke indicated his impression that ‘the incidence of “male rape” appears to be low, with just 13 convictions in 1990’.^109^ This contradicted the conclusions of a meeting three years earlier between Home Office staff, psychiatrists and Metropolitan Police representatives. The police used this meeting to clarify that ‘about 4 reports a month’ were being received from ‘men alleging they had been raped’, as well as the fear they encountered from victims that they would be disbelieved.^110^ The psychiatrists in attendance, who were ‘conducting the only research on male rape in this country’ at the time, confirmed this fear of disbelief and dismissal as a major barrier to men reporting their rapes to the police.^111^ Clarke had not been Home Secretary in 1987 and may have been unaware of this report.^112^ Many Home Office staff, however, were likely to have been aware of the report and certainly had access to it. Either way, the Home Office decision not to change the law overlooked what appeared to be a growing number of cases and advice from the Metropolitan Police Service and the Institute of Psychiatry that minimizing the effects of male rape was a significant barrier to men self-reporting. This was despite the draft Home Office letter referring in its first sentence to the ‘considerable public concern that the law does not adequately recognise the trauma suffered by victims of so-called “male rape”’.^113^

Inaction from the Home Office prompted Cohen to change tack. In 1992 he introduced a Private Member’s Bill to the House of Commons which, in his own words, ‘would make male rape a specific criminal offence for the first time. It would do that simply by changing the definition of the victim from “woman” to “person” and it would thus allow men to be considered in law to be the victims of rape’.^114^ This was part of a broader package of reforms to rape legislation proposed by Cohen which sought to make marital rape a criminal offence and expand rights for victims of rape.^115^ Once again, ministers were unconvinced. Both the Home Secretary and the Attorney General were concerned that changing the legal definition of rape to include male victims would prove to be a divisive legislative move. Writing to Clarke, Attorney General Nicholas Lyell argued that ‘we should avoid a controversial agenda for sexual offences law reform’, adding that he consequently agreed ‘that the [Cohen] Bill should be blocked’.^116^ This letter was written just three days after a man had been raped by two men in Cohen’s constituency.^117^ This case was not mentioned in the correspondence between Cohen and the Home Office (despite Cohen mentioning it in the Commons), but it serves as a reminder that these were live issues with real impacts as debates about changing the law stalled.^118^ Cohen’s Sexual Offences (Amendment) Bill failed to get a Second Reading in the House of Commons, after being objected to six times.^119^

In April 1994, the Government was still insisting that it had ‘no plans’ to create an offence of male rape.^120^ However, this blanket denial obscured the fact that the Government had quietly adopted the logic of the earlier Cohen Bill by amending the Sexual Offences Act 1956 to extend the definition of rape to include male victims.^121^ It did so by adding a clause stating that, in addition to forced vaginal rape, a man commits rape if he ‘has anal intercourse with a woman or with another man who (in either case) at the time of the act does not consent to it’.^122^ As a result, the Criminal Justice and Public Order Act 1994 cemented the fact that ‘It is an offence for a man to rape a woman or another man’.^123^

The debates over the Criminal Justice and Public Order Act begin to reveal why the introduction of male rape legislation was considered so ‘controversial’. The persistent lack of visibility, which groups like Survivors sought to challenge, led some lawmakers to question the necessity of such legislation. Lord Ponsonbury of Shulbrede, the Labour hereditary peer who introduced the amendment which ultimately succeeded in legislating against male rape, was at pains to convince fellow members of the House of Lords that, despite ‘the idea of male rape [being] unthinkable to many of your Lordships, as it is to me … it has become clear from the newspapers that it does happen’.^124^ For others, such as Conservative peer Earl Ferrers, the ‘central question is whether it is right to call those crimes "rape”’. For those who shared his view, ‘rape is well [understood] as a particular crime – one that can be committed only by a man who has intercourse with a woman without her consent’.^125^ To change the definition would complicate what they saw as a common sense piece of legislation. Even Conservative peer Lord Swinfen, who supported the amendment, was ‘not sure that “male rape” or “rape” is the right description of the crime’, wondering instead whether ‘non-consensual buggery is perhaps more accurate’.^126^

There was also a widespread concern that ‘male rape’ would be seen as a gay issue. In his attempt to convince his colleagues that male rape was prevalent enough to warrant legislation, Lord Ponsonbury also argued that ‘male rape should not be regarded as a homosexual offence’. Instead, he pointed out that many offenders were heterosexual, and that male rape was ‘part of a larger pattern of violent attacks’.^127^ Data on the calls to the Survivors helpline was cited in the Commons to back this up, demonstrating a roughly even split of heterosexual and homosexual victims.^128^ Importantly, though, the debates reveal that – unlike those concerning reducing the age of consent for gay men, which occurred in parallel – very few objections were raised to the introduction of an offence of male rape. Once parliamentarians had been able to discuss the issue of male rape they had demonstrated, perhaps to the surprise of some ministers, that they did not consider such legislative reform particularly controversial.

Medical research, particularly psychiatric studies, proved influential in assuaging the concerns of British legislators. As we have already seen, psychiatric research was a major factor informing ministry-level thinking about raped men, and such studies were frequently drawn upon in parliamentary debates over whether to legislate against male rape. Such research anchored the passionate call for a new clause of the Criminal Justice Act criminalizing male rape made by Alan Howarth, Conservative MP for Stratford-on-Avon. Howarth argued that such a clause was ‘needed because the present situation is plainly inequitable’.^129^ He then quoted several psychiatric and medical studies which had shown the traumatic impact of rape on men. These included an article by Michael King in the *British Medical Journal* which concluded that rape trauma syndrome ‘occurs in both men and women’, Zsuzsanna Adler’s research explaining low levels of male survivors reporting rape to the police, and Gillian Mezey’s suggestion that prevalence data being reported in 1994 were ‘the tip of an iceberg’.^130^ Research by other medical experts, notably clinical medics and general practitioners, influenced these debates, but it was psychiatric research which proved most effective at reaching the attention of parliamentarians, ministers and civil servants considering legislating against male rape.

The new psychiatric research on men’s rape trauma proved such a persuasive tool for two main reasons. First, this literature rendered visible the mental injuries which men sustained in the process of being raped. Rape myths, underpinned by dominant cultural assumptions about masculinity, led to a belief that male bodies were sexually inviolable or that their bodies would display the physical injuries of resistance. Trauma-informed psychiatric research revealed that unwanted sex was not only possible without physical force, but that it proved deeply damaging to male psyches. Second, ideas about masculinity and trauma were shifting in late twentieth-century Britain, and by the 1990s images of masculinity were becoming less rigid. In particular, combatants returning from conflicts such as the Falklands War found British society more receptive to their resulting mental anguish than many of their predecessors had, in part due to developing ideas of PTSD as well as the recognition of the widespread mental impact of the First World War.^131^ Of course, this did not mean that displays or discussion of male psychic unease became universally possible. Trauma provided a new medicalized language but, as Helen Parr has argued, displays and discussions of grief remained heavily gendered and were widely avoided by men affected by the Falklands War.^132^ Physical injuries continued to have more purchase over public sympathies (think, for example, of the coverage of Simon Weston’s significant burn injuries, which Lucy Robinson notes are ‘generally presented as physical rather than traumatic’), but the emergent language of trauma provided greater visibility, and sympathy, for men with PTSD.^133^ It was in this shifting landscape of public and medical understandings of male emotions that calls to legislate against male rape found cultural and political purchase.

## CONCLUSION

The change to the law on male rape was one of several redefinitions of rape in law across the United Kingdom around the turn of the century. The provision allowing a man to legally rape his wife was removed following decades of feminist campaigning in 1994.^134^ Two years later, a 1996 Crown Court ruling established the precedent that forced penetration of a trans woman’s ‘artificial vagina can, in law, constitute rape’.^135^ Forced fellatio was included in rape law in 2003.^136^ Male rape became a legal category in Northern Ireland in 2008 when rape legislation was brought into line with English and Welsh law, with the Sexual Offences (Scotland) Act doing the same in 2009.^137^

By the mid-1990s, levels of support available to male survivors had increased. In Leicester, for example, First Step was established in May 1997 to provide counselling to male survivors (both in person and over their telephone helpline), as well as facilitating relevant professionals with training about male survivors of sexual abuse.^138^ The group worked closely with local police, offering training and collaborating on the resources the police made available to male survivors. As a result, Leicestershire Constabulary produced ‘a guide for men reporting rape’ and offered male-specific support in their rape victim support unit.^139^ First Step was one of a patchwork of local services which accompanied the more nationally-oriented Survivors group.

In Birmingham, the Birmingham Rape Crisis and Research Centre had transformed into the Rape and Sexual Violence Project (RSVP) between 1993 and 1995. RSVP literature began using more gender-neutral language, sometimes talking of ‘people’ instead of ‘women’ as a standard collective noun when discussing their service users. Explicitly, RSVP noted in its Annual Report in 1995 that ‘the introduction of the Public Order and Criminal Justice Act 1994 [in which] rape of men was recognised’, coupled with the fact that there were ‘very few services for men who have been raped’, meant that the Project would begin to engage with male survivors. Volunteers with RSVP were given training on working with men as part of ‘Equal Opportunities issues’ alongside ‘Black women survivors’, ‘lesbian survivors’ and ‘women with disabilities’.^140^ This included taking calls from men to the RSVP helpline. By the mid-1990s, men made up nearly 13% of such calls (women remained the overwhelming majority of callers at over 70%).^141^

Such figures are an important reminder that a history of male survivors – and the wider political call for gender-inclusive legislation and service provision – must be a feminist enterprise, one which brings male survivors into view without diminishing or undermining the plight of women who have been subjected to acts of sexual violence. It is also a reminder of the reason that many rape crisis centres have continued to deliver services to women and been reluctant to engage with men. In some areas, uptake of specialist services for men has been low while demand for rape crisis services by women has grown. One paediatrician lamented the fact that male victims of rape were ‘marginalize[d] … completely’, but contrasted this with her own local context:

if you’re a man who has been raped in Taunton, you can get therapy within about four weeks because there’s a specialist clinic and it’s not got a lot of take up and it’s well funded. Whereas if you are a teenage girl who’s been raped, you have to wait at least nine months if not a year.^142^

As this article has discussed, the reasons that so few male survivors seek support or report their experiences are legion. Being attentive to these factors and addressing them both historically and in contemporary policymaking must not be at the expense of other survivors.

In exploring the differing levels of visibility male survivors of rape experienced across the late twentieth century, this article has also begun to explain the rapid shifts which rape support staff had witnessed. To be sure, the change in the law made a significant difference: whether through greater awareness or new statutory obligations attached to their funding, rape support services began to provide more support for men following the change in the law. However, it was not simply legal recognition of male rape which resulted in greater awareness. Psychiatric understandings of male survivors produced by a new generation of forensic psychiatric researchers brought men who were subjected to sexual violence more squarely into the view of policymakers in England and Wales.

Nonetheless, despite the increased visibility of raped men across the late twentieth century discussed in this article, these changes were not straightforward or permanent. The 1990s saw an increase in support groups, psychiatric studies and policymaking focused on men who had been raped, but Mezey’s pessimism about the impact of her studies and the longevity of their results was to some extent well placed. Indeed, in 1999, five years after the legal recognition of male rape, queer activists launched an advertising campaign designed to raise awareness of rape among gay men.^143^

But however short-term, the increasing levels of visibility and understanding of male survivors of rape in late twentieth-century Britain reveal much about the ways in which ideas of masculinity were changing. During this period, normative masculinity was reckoning with increased knowledge and examples of trauma. New psychiatric models of trauma, particularly PTSD, rendered traumatized men more legible. From traumatized combatants in the Falklands to men who had been raped, men were able to articulate unseen mental injury in newly resonant ways. The developing psychiatric literature on trauma not only transformed how victims’ experiences were seen and understood (including in legal terms); it helped to change gendered notions of sexual victimhood.

## Footnotes

1 ‘First man jailed for male rape’, 9 June 1995, http://news.bbc.co.uk/onthisday/low/dates/stories/june/9/newsid_2500000/2500803.stm [accessed 31/08/2022].

2 A. D. Harvey, ‘Prosecutions for Sodomy in England at the Beginning of the Nineteenth Century’, *Historical Journal* 21: 4, 1978, pp. 939–48, 941; Joanna Bourke, *Loving Animals: On Bestiality, Zoophilia and Post-human Love*, London, 2020, p. 42.

3 H. G. Cocks, ‘Safeguarding Civility: Sodomy, Class and Moral Reform in Early Nineteenth-Century England’, *Past and Present* 190: 1, 2006, pp. 121–46.

4 Cocks, ‘Safeguarding Civility’, p. 142; Harvey, ‘Prosecutions for Sodomy in England’, p. 941.

5 See Joanna Bourke, *Disgrace: Global Reflections on Sexual Violence*, London, 2022, p. 20.

6 See Ian Barnard, ‘Sexual Assaults, Queer Panics: Gemma Watts and Reynhard Sinaga’, in *The Routledge Handbook of Queer Rhetoric*, ed. Jacqueline Rhodes and Jonathan Alexander, New York, 2022.

7 Male Survivors Partnership, Lime Culture and NHS England, ‘Supporting male victims/survivors accessing a sexual assault referral centre’, NHS England Official Report, February 2022, p. 5 and n2.

8 Susan Brownmiller, *Against Our Will: Men, Women and Rape*, New York, 1975, p. 15, pp. 257–68.

9 See, for example, Joanna Bourke, *Rape: A History from 1860 to the Present*, London, 2007, pp. 238–48, and Joanna Bourke, ‘Military Sexual Trauma: Gender, Military Cultures, and the Medicalization of Abuse in Contemporary America’, *Journal of War & Culture Studies* 15: 1, 2022, pp. 86–105.

10 Bourke, *Disgrace*, pp. 74–82.

11 Bourke, *Rape*, p. 247.

12 Deana Heath, ‘Torture, the State, and Sexual Violence against Men in Colonial India’, *Radical History Review* 126, 2016, pp. 122–33, 124–5.

13 See, for example, Garthine Walker, ‘Rape, Acquittal and Culpability in Popular Crime Reports in England, c. 1670-1750’, *Past and Present* 220: 1, 2013, pp. 115–42; Laura Lammasniemi, ‘Trafficking, Rape, or Deceptive Sex? A Historical Examination of Procurement Offences in England’, *Social and Legal Studies* 32: 4, 2023, pp. 449–518; and Adrian Williamson, ‘The Law and Politics of Marital Rape in England, 1945–1994’, *Women’s History Review* 26: 3, 2017, pp. 382–413.

14 Heath, ‘Sexual Violence against Men’, p. 125.

15 Bourke, *Rape*, p. 247.

16 Orna Alyagon Darr, *Plausible Crime Stories: The Legal History of Sexual Offences in Mandate Palestine*, Cambridge, 2019, chapter 2; Heath, ‘Sexual Violence against Men’.

17 ‘Forum ’91’, *Gay Scotland* 59, 1991, p. 14.

18 Belfast Rape Crisis Centre, Library of Birmingham, MS1579/2/11/5/10b.

19 Clare Leon, ‘Sexual Offences in Ireland 1994–1997’, *British Society of Criminology* 3, June 2000: Selected Papers from the 1999 British Criminology Conference, Liverpool, n.p. Leon attributes this rise to the impact of media coverage of sexual assaults throughout 1995.

20 Bourke, *Rape*, p. 212.

21 Siobhan Weare, ‘“Oh you’re a guy, how could you be raped by a woman, that makes no sense”: Towards a Case for Legally Recognising and Labelling ‘Forced-to-penetrate’ Cases as Rape’, *International Journal of Law in Context* 14:1, 2018, pp. 110–31.

22 Ruth Beecher, ‘Children, Sexual Abuse and the Emotions of the Community Health Practitioner in England and Wales, 1970–2000’, *Journal of the History of Medicine and Allied Sciences* 78: 4, 2023, pp. 365–80; see pp. 373 and 365 respectively. See also Lucy Delap, ‘“Disgusting Details Which Are Best Forgotten”: Disclosures of Child Sexual Abuse in Twentieth-Century Britain’, *Journal of British Studies* 57: 1, 2018, pp. 79–107.

23 See Margaretta Jolly, *Sisterhood and After: An Oral History of the UK Women’s Liberation Movement, 1968–Present*, Oxford, 2019, pp. 12, 95, 170–4; Williamson, ‘The Law and Politics of Marital Rape’, esp. p. 391. See also Sue Bruley, ‘Women’s Liberation at the Grass Roots: A View from some English Towns, c. 1968–1990’, *Women’s History Review* 25: 5, 2016, pp. 723–40, esp. 731.

24 Minutes of BRCRC, meeting held Tuesday 29^th^ March 1983, item 5, Library of Birmingham: MS 1579/2/5/1/3.

25 There are no mentions of bisexuality or trans individuals in the BRCRC papers from this period.

26 Lucy Delap, ‘Rethinking Rapes: Men’s Sex Lives and Feminist Critiques’, *Contemporary British History* 36: 2, 2022, pp. 253–76.

27 Delap, ‘Rethinking Rapes’.

28 Terry Gillespie, ‘Rape Crisis Centres and Male Rape: A Face of the Backlash’, in *Women, Violence and Male Power*, ed. Marianne Hester, Liz Kelly and Jill Radford, Buckingham, 1995, p. 157.

29 Gerrilyn Smith interviewed by Ruth Beecher, 12 May 2021, interview currently held by Ruth Beecher (hereafter RB).

30 Gillian Mezey and Michael King, ‘The Effects of Sexual Assault on Men: A Survey of 22 Victims’, *Psychological Medicine* 19, 1989, pp. 205–9, 208.

31 See Adrian W. Coxell and Michael B. King, ‘Male Victims of Rape and Sexual Abuse’, *Sexual and Marital Therapy* 11: 3, 1996, pp. 297–308; F.C. Hickson, P. M. Davies, A.J. Hunt, P. Weatherburn, T. J. McManus, A. P. Coxon, ‘Gay Men as Victims of Nonconsensual Sex’, *Archives of Sexual Behaviour* 23: 3, June 1994, pp. 281–94.

32 Michael B. King, ‘Male Rape’, *British Medical Journal* 301: 6765, 15 December 1990, pp. 1345–6.

33 Friend, London School of Economic Archives: HCA/CHE2/12/13. See Teri Chettiar, ‘Counselling for Connection: Making Queer Relationships during Britain’s Sexual Revolution’, *Medical Humanities* 49, 2023, pp. 182–92.

34 Gerrilyn Smith interviewed by Ruth Beecher 12 May 2021, RB. See also Richie J. McMullen, *Male Rape: Breaking the Silence on the Last Taboo*, London, 1990, p. 127.

35 Gerrilyn Smith interviewed by Ruth Beecher 12 May 2021, RB.

36 Bourke, *Rape*, p. 240.

37 Lisa Power, ‘Voices in my Ear’, in *Radical Records: Thirty Years of Lesbian and Gay History, 1957–1987*, ed. Bob Cant and Susan Hemmings, London, 2010, p. 89; Bishopsgate Institute Library (hereafter BIL): SB/1/2/3.

38 Legal log 21/3/92, BIL: SB/5/3/4.

39 Legal log 9/2/92, BIL: SB/5/3/4.

40 Legal log 30/3/92 BIL: SB/5/3/4.

41 On GLAD see Warren Blumenfeld, ‘Gay Life in Merry Old England’, *Gay Community News*, 7: 18, 24 November 1979, p. 9, and Ann Darnborough, *The Sex Directory: A Guide to Sexual Problems and Where to Go for Help*, Cambridge, 1988, p. 163. On GALOP see Sarah Lamble, ‘Queer Investments in Punitiveness: Sexual Citizenship, Social Movements and the Expanding Carceral State’, in *Queer Necropolitics*, ed. Jin Haritaworn, Adi Kuntsman and Silvia Posocco, Oxford, 2014, pp. 158–9. My thanks to Lisa Power and Richard Desmond, both former Switchboard volunteers, for helping to clarify some facts about GLAD.

42 Legal log 3/3/92, BIL: 5/3/4.

43 McMullen, *Male Rape*, p. 9.

44 I am grateful to Alastair Hilton and Jim MacSweeney for sharing their memories of this book.

45 Legal log 21/3/92, BIL: SB/5/3/4.

46 Letter from Barbara Wilding to Pamela Johnson, 5 November 1992, The National Archives (hereafter TNA): HO 287/4378.

47 Ernest Woollett, ‘Men Don’t Get Raped!’, *Clinical Psychology Forum* 64, February 1994, pp. 26–7, 26.

48 ‘Survivors Sheffield’, *Men for Change* 18, Winter 1991, p. 10.

49 McMullen, *Male Rape*, p. 125.

50 Hansard House of Commons Debates 12 April 1994, vol 241 c177 (hereafter HC Deb); Jayne Lizbeth Walker, ‘A Study of Male Rape Survivors’, Unpublished PhD Thesis: University of Central Lancashire, 2004, p. 65.

51 On the anti-sexist men’s movement in Britain see Lucy Delap, ‘Feminism, Masculinities and Emotional Politics in Late Twentieth Century Britain’, *Cultural and Social History* 15: 4, 2018, pp. 571–93.

52 ‘Reading Survivors’, *Men for Change* 18, Winter 1991, p. 10.

53 ‘F.S.C.S.A.M CONTRIBUTORS AND WORKSHOP FACILITATORS’, Liz Kelly uncatalogued papers, London Metropolitan University (hereafter LMU).

54 Mezey and King, ‘The Effects of Sexual Assault on Men’, p. 208.

55 This policy changed on 9^th^ November 1990; see letter from Barbara Wilding to Pamela Johnson, 5 November 1992, TNA: HO 287/437

56 Legal log, 3/3/92, BIL: SB/5/3/4.

57 Matt Cook, ‘“Archives of Feeling”: the AIDS Crisis in Britain 1987’, *History Workshop Journal* 83, Spring 2017, pp. 51–78.

58 Richard Hillman, Nigel O’Mara, David Tomlinson and J. R. William Harris, ‘Adult Male Victims of Sexual Assault: An Underdiagnosed Condition’, *International Journal of STD & AIDS* 2: 1, 1991, pp. 22–4.

59 Letter from Barbara Wilding to Pamela Johnson, 5 November 1992, TNA: HO 287/4378.

60 Richard Hillman, David Tomlinson, A. McMillan, P. D. French and J. R. William Harris, ‘Sexual Assault of Men: A Series’, *Genitourinary Medicine* 66, 1990, p. 247.

61 S. Dougan, B. G. Evans, N. MacDonald, D. J. Goldberg, O. N. Gill, K. A. Fenton and J. Elford, ‘HIV in Gay and Bisexual Men in the United Kingdom: 25 Years of Public Health Surveillance’, *Epidemiology and Infection* 136: 2, 2008, pp. 145–56, 150.

62 Cook, ‘Archives of Feeling’, p. 72. Note that this is the total death toll from the start of the epidemic.

63 Matt Cook, ‘AIDS, Mass Observation, and the Fate of the Permissive Turn’, *Journal of the History of Sexuality* 26: 2, 2017, pp. 239–72, 270.

64 Cook, ‘Fate of the Permissive Turn’, p. 262.

65 Cook, ‘Fate of the Permissive Turn’, p. 265; HL Deb 20 June 1994 vol 556 c64.

66 Michael Savage, ‘Anger over new ministerial job for MP accused of asking if alleged Pincher victim was gay’, *Guardian*, 10 July 2022, https://www.theguardian.com/politics/2022/jul/10/anger-over-new-minister-job-for-mp-accused-of-asking-if-alleged-pincher-victim-was-gay-sarah-dines [accessed 14/06/2023]. For a succinct discussion of the concept of ‘rapability’, see Holly Henderson, ‘Feminism, Foucault, and Rape: A Theory and Politics of Rape Prevention’, *Berkeley Journal of Gender, Law and Justice* 22, 2007, pp. 225–53, 241.

67 Clare Dyer, ‘Rape law helps male victims’, *Guardian*, 12^th^ July 1994, p. 2.

68
*Witness Seminar: The Criminalisation of HIV Transmission in the UK*, ed. Emily Jay Nicholls and Marsha Rosengarten, Disentangling European HIV/AIDS Policies: Activism, Citizenship and Health (EUROPACH), 2019.

69 R v Dica [2004] EWCA Crim 1103; [2004] QB 1257 (CA), and R v Konzani [2005] EWCA Crim 706; [2005] 2n Cr App R 14.

70 Cook, ‘Fate of the Permissive Turn’, pp. 266–7; Bourke, *Rape*, p. 240.

71 Patricia H. Allderidge, ‘Historical Notes on the Bethlem Royal Hospital and the Maudsley Hospital’, *Bulletin of the New York Academy of Medicine* 47: 12, December 1971, pp. 1537–46, 1545.

72 Gillian Mezey interviewed by author, 6 May 2022, Birkbeck BiRD.

73 Joanna Bourke, ‘Sexual Violence, Bodily Pain, and Trauma: A History’, *Theory, Culture, Society* 29: 3, 2012, pp. 25–51, see pp. 43–4; Gill Mezey, ‘Hospital Based Rape Crisis Programmes: What can the American Experience Teach Us?’, *Bulletin of the Royal College of Psychiatrists* 11: 2, 1987, pp. 49–51.

74 Lucy Robinson, ‘Soldiers’ Stories of the Falklands War: Recomposing Trauma in Memoir’, *Contemporary British History* 25: 4, 2011, pp. 569–89, 569.

75 See, for example, Gillian C. Mezey and Pamela J. Taylor, ‘Psychological Reactions of Women Who Have Been Raped: A Descriptive and Comparative Study’, *British Journal of Psychiatry* 152: 3, 1988, pp. 330–9.

76 Gillian Mezey interviewed by author, 6 May 2022, Birkbeck BiRD.

77 Gillian Mezey interviewed by author, 6 May 2022, Birkbeck BiRD.

78 Gillian Mezey and Michael King, ‘Male Victims of Sexual Assault’, *Medicine, Science and the Law* 27: 2, 1987, pp. 122–124.

79 A Google Scholar search on 22 February 2024 shows Mezey and King (1987) as having sixty-six citations compared to 304 for Mezey and King (1989).

80 ‘First Standing Conference on Sexual Abuse of Men’, Liz Kelly uncatalogued papers, LMU.

81
*Gay Liberation Front Manifesto*, London, 1971, p. 5.

82 Michael King and Annie Bartlett, ‘British Psychiatry and Homosexuality’, *British Journal of Psychiatry* 175: 2, August 1999, pp. 106–113, 110.

83 Mezey and King, ‘The Effects of Sexual Assault on Men’, p. 205.

84 Jessica A. Turchik and Katie M. Edwards, ‘Myths About Male Rape: a Literature Review’, *Psychology of Men and Masculinity* 13: 2, 2012, pp. 211–226; Arthur N. Gilbert, ‘Buggery and the British Navy, 1700–1861’, *Journal of Social History* 10: 1, 1976, pp. 72–98. Alan Hollinghurst’s first novel *The Swimming Pool Library* featured a rape scene in an imagined nineteenth-century boarding school; Alan Hollinghurst, *The Swimming Pool Library*, London, 2015 [1988], pp. 156–7.

85 Mezey and King, ‘The Effects of Sexual Assault on Men’, p. 207.

86 Mezey and King, ‘The Effects of Sexual Assault on Men’, p. 208.

87 See, for example, Hillman, Tomlinson, McMillan, French and Harris, ‘Sexual Assault of Men’, esp. n. 5.

88 Cook, ‘Archives of Feeling’, esp. pp. 51, 62–4.

89 See, for example, ‘Raped by gays’, *The Sun*, 22 February 2001; Nicholas A. Groth and Ann W. Burgess, ‘Male Rape: Offenders and Victims’, *The American Journal of Psychiatry* 137: 7, 1980, pp. 806–10.

90 On male rape myths see Scott M. Walfield, ‘“Men Cannot Be Raped”: Correlates of Male Rape Myth Acceptance’, *Journal of Interpersonal Violence* 36: 13/14, 2021, pp. 6391–417.

91 Bourke, *Rape*, p. 245. For studies which point to an erectile response being triggered by acts of sexual violence, see p. 479 n. 128.

92 Mezey and King, ‘The Effects of Sexual Assault on Men’, p. 207.

93 Mezey and King, ‘The Effects of Sexual Assault on Men’, p. 207.

94 Mezey and King, ‘The Effects of Sexual Assault on Men’, p. 207.

95 Memorandum, List A and List B, 16/7/87, TNA: HO 287/4378.

96 The book in question is *Male Victims of Sexual Assault*, ed. Gillian C. Mezey and Michael B. King, Oxford, 1992.

97 Memorandum from Sue Adler to Kevin Sheehan, 3 April 1989, and associated documents, TNA: HO 287/4378.

98 The Effects of Sexual Assault on Men: A Survey of 22 Victims, TNA: HO 287/4378.

99 Gillian Mezey interviewed by author, 6 May 2022, Birkbeck BiRD.

100 Gillian Mezey interviewed by author, 6 May 2022, Birkbeck BiRD.

101 Chris Gregory, ‘Former police college site set for 250-home scheme’, *Basingstoke Gazette*, published online 4 June 2015.

102 Gillian Mezey interviewed by author, 6 May 2022, Birkbeck BiRD.

103 Janine Di Giovanni, ‘Society’s new forgotten victims’, *Sunday Times*, 17 May 1992, pp. 1–2.

104 HC Deb 18 May 1992 vol 208 c3.

105 Andrew Penman, ‘Male rape, the crime that dare not speak its name’, *Today*, 15 May 1992.

106 A similar pattern is observed in the Republic of Ireland in Leon, ‘Sexual Offences in Ireland 1994–1997’.

107 Adrian Williamson, ‘The Law and Politics of Marital Rape’, p. 401.

108 TNA: HO 287/4378.

109 Draft letter to the Lord Privy Seal for signature by the Home Secretary, TNA: HO 287/4378.

110 Four reports a month would mean that the Metropolitan Police Service alone was receiving c. 48 complaints a year, more than double the number cited by the draft Clarke letter.

111 ‘Note for file: meeting on 13.8.1987’ and ‘Those attending male rape meeting on 13 August 1987’, TNA: HO 287/4378.

112 Clarke held several ministerial roles in 1987: Paymaster-General and Employment Minister, Chancellor of the Duchy of Lancaster, and Department of Trade and Industry Minister, before becoming Secretary of State for Health in July 1988.

113 Draft letter to the Lord Privy Seal for signature by the Home Secretary, TNA: HO 287/4378.

114 HC Deb 30 April 1993 vol 223 c1260.

115 HC Deb 28 October 1992 vol 212 cc1021–3.

116 Letter from the Attorney General to the Home Secretary, 29 October 1992, TNA: HO 287/4378.

117 Helen Johnstone, ‘Man raped in his bedsit’, *The Times*, 28 October 1992, p. 6.

118 HC Deb 28 October 1992 vol 212 c1021.

119 HC Deb 11 December 1992 vol 215 c1174; HC Deb 12 February 1993 vol 218 c1273; HC Deb 23 April 1993 vol 223 c693; HC Deb 30 April 1993 vol 223 c1326; HC Deb 14 May 1993 vol 224 c1105; HC Deb 02 July 1993 vol 227 c1286.

120 HC Deb 28 April 1994 vol 242 c244W.

121 HC Deb 12 April 1994 vol 241 c172.

122 HC Deb 12 April 1994 vol 241 c172.

123 Criminal Justice and Public Order Act 1994, Section 142.1.

124 HL Deb 20 June 1994 vol 556 c63.

125 HL Deb 20 June 1994 vol 556 c65.

126 HL Deb 20 June 1994 vol 556 c66.

127 HL Deb 20 June 1994 vol 556 c64.

128 HC Deb 12 April 1994 vol 241 c177.

129 HC Deb 12 April 1994 vol 241 c176.

130 HC Deb 12 April 1994 vol 241 c177.

131 Robinson, ‘Soldiers’ Stories’; Tracey Loughran, ‘Shell Shock, Trauma, and the First World War: The Making of a Diagnosis and Its Histories’, *Journal of the History of Medicine and Allied Sciences* 67: 1, 2012, pp. 94–119.

132 Helen Parr, ‘Representations of Grief and the Falklands War’, in *Emotions, Politics and War*, ed. Linda Åhäll and Thomas Gregory, London, 2015, p. 160. See also Thomas Dixon, ‘Weeping in Space: Tears, Feelings and Enthusiasm in Eighteenth-Century Britain’, in *Spaces for Feeling: Emotions and Sociabilities in Britain, 1650–1850*, ed. Susan Broomhall, London, 2015, for a longer view of the history of gendered (male) emotions.

133 Robinson, ‘Soldiers’ Stories’, p. 578.

134 Williamson, ‘The Law and Politics of Marital Rape’, p. 401.

135
*R. v Matthews*, Reading Crown Court, unreported, 28 October 1996.

136 The Sexual Offences Act 2003 Section 1 identified a rapist as ‘intentionally penetrating the vagina, anus or mouth of another person (B) with his penis’.

137 The Sexual Offences (Northern Ireland) Order 2008; The Sexual Offences (Scotland) Act 2009. The Order and Act respectively phrase the relevant sections (each section 1) differently, but the points of law – that a male person could be the victim of a rape – are the same.

138 ‘Partnership Success’, *first step* 1: 4, November 1998, p. 1.

139 Leicestershire Constabulary, ‘A guide for men reporting rape’, Record Office for Leicestershire, Leicester and Rutland: 364.1532 Eph.

140 Rape & Sexual Violence Project Report for 1993–4 1994–5, p. 13, Library of Birmingham Archives: MS1579/2/16/2/1.

141 Rape & Sexual Violence Project Report for 1993–4 1994–5, p. 8, Library of Birmingham Archives: MS1579/2/16/2/1. Silent calls made up the remainder.

142 Michelle Cutland interviewed by Ruth Beecher, 28 September 2021, RB.

143 ‘5,000 gay men raped every year’, *The Pink Paper*, 14 May 1999, n.p; BIL: GMFA/39. GMFA was co-founded by Martin Dockrell, the activist and survivor who had co-founded Survivors.

## Author Biography

**George J. Severs** is a Postdoctoral Researcher at the Geneva Graduate Institute in Switzerland. He is a historian of sexual health, sexual violence and HIV/AIDS. His first book, *Radical Acts: HIV/AIDS Activism in Late Twentieth-Century England*, will be published by Bloomsbury Academic in 2024.

## Notes

This article was written when I was a Postdoctoral Research Fellow on the Sexual Harms and Medical Encounters (SHaME) project at Birkbeck, University of London and funded by the Wellcome Trust (Grant ID 205378/Z/16/Z). The Geneva Graduate Institute Library generously covered the cost of making this article available via Open Access, for which I thank them. Switchboard generously granted me permission to their archives and agreed to my use of the quotes published in this article. I am grateful to Ruth Beecher, Sam Cole, Matt Cook, Lucy Delap, Lauren Gutterman, the reviewing *History Workshop Journal* editors (especially Kate Hodgkin, who worked beyond the call of duty), and attendees of the University of Birmingham’s History of Medicine and Health Development Seminar and the History, Classics and Archaeology departmental seminar at Birkbeck for feedback on earlier versions of this article. For sharing and helping me to access sources, my thanks to Ruth Beecher, Lucy Delap and James Morris.

